# Characterisation of Immune and Neuroinflammatory Changes Associated with Chemotherapy-Induced Peripheral Neuropathy

**DOI:** 10.1371/journal.pone.0170814

**Published:** 2017-01-26

**Authors:** Preet G. S. Makker, Samuel S. Duffy, Justin G. Lees, Chamini J. Perera, Ryan S. Tonkin, Oleg Butovsky, Susanna B. Park, David Goldstein, Gila Moalem-Taylor

**Affiliations:** 1 School of Medical Sciences, University of New South Wales, Sydney, New South Wales, Australia; 2 Ann Romney Center for Neurologic Diseases, Brigham and Women's Hospital, Harvard Medical School, Boston, Massachusetts, United States of America; 3 Brain and Mind Centre, Sydney Medical School, The University of Sydney, New South Wales, Sydney, Australia; 4 Department of Medical Oncology, Prince of Wales Hospital, New South Wales, Randwick, Australia; Boston Children’s Hospital and Harvard Medical School, UNITED STATES

## Abstract

Chemotherapy-induced peripheral neuropathy (CIPN) and associated neuropathic pain is a debilitating adverse effect of cancer treatment. Current understanding of the mechanisms underpinning CIPN is limited and there are no effective treatment strategies. In this study, we treated male C57BL/6J mice with 4 cycles of either Paclitaxel (PTX) or Oxaliplatin (OXA) over a week and tested pain hypersensitivity and changes in peripheral immune responses and neuroinflammation on days 7 and 13 post 1^st^ injection. We found that both PTX and OXA caused significant mechanical allodynia. In the periphery, PTX and OXA significantly increased circulating CD4+ and CD8+ T-cell populations. OXA caused a significant increase in the percentage of interleukin-4+ lymphocytes in the spleen and significant down-regulation of regulatory T (T-reg) cells in the inguinal lymph nodes. However, conditional depletion of T-reg cells in OXA-treated transgenic DEREG mice had no additional effect on pain sensitivity. Furthermore, there was no leukocyte infiltration into the nervous system of OXA- or PTX-treated mice. In the peripheral nervous system, PTX induced expression of the neuronal injury marker activating transcription factor-3 in IB4+ and NF200+ sensory neurons as well as an increase in the chemokines CCL2 and CCL3 in the lumbar dorsal root ganglion. In the central nervous system, PTX induced significant astrocyte activation in the spinal cord dorsal horn, and both PTX and OXA caused reduction of P2ry12+ homeostatic microglia, with no measurable changes in IBA-1+ microglia/macrophages in the dorsal and ventral horns. We also found that PTX induced up-regulation of several inflammatory cytokines and chemokines (TNF-α, IFN-γ, CCL11, CCL4, CCL3, IL-12p70 and GM-CSF) in the spinal cord. Overall, these findings suggest that PTX and OXA cause distinct pathological changes in the periphery and nervous system, which may contribute to chemotherapy-induced neuropathic pain.

## Introduction

Chemotherapy-induced peripheral neuropathy (CIPN) is a dose-limiting neurotoxic effect of chemotherapy. It is a common cause for early cessation of cancer treatment and affects 30–70% of patients receiving chemotherapy [1] depending upon the particular anticancer drug or drug combination, as well as the dosing regimen [2, 3]. Patients with CIPN experience sensory abnormalities including symptoms of neuropathic pain, such as paraesthesia and dysesthesia (abnormal sensations including tingling, numbness and pins and needles), allodynia (pain due to a stimulus that does not normally provoke pain) and hyperalgesia (increased pain from a stimulus that normally provokes pain) [4, 5]. Such symptoms can persist well beyond the discontinuation of treatment, leading to long-lasting disability. Symptoms initially develop in the hands and feet, followed by proximal progression in a *stocking and glove* distribution and can exhibit a *coasting* effect, whereby there is a delay between onset of chemotherapy and onset of neuropathy [6]. Anticancer drugs that commonly induce CIPN include platinum and anti-tubulins/spindle compounds. These drugs may cause neurotoxicity by affecting different aspects of the nervous system. For example, oxaliplatin (OXA), a platinum-based DNA-intercalating agent, induces neuronal damage and axonal degeneration through DNA damage [7]. Alternatively, paclitaxel (PTX), an anti-tubulin drug, causes microtubule stabilisation resulting in distal axonal degeneration in the peripheral nervous system (PNS), secondary demyelination and nerve fibre loss [8, 9].

Neuroinflammation has been implicated in several neuropathic pain models [10, 11] including traumatic nerve injury [12] and diabetic neuropathy [13], and its role in CIPN pathology is becoming increasingly evident. Chemotherapy drugs can penetrate the blood-nerve barrier, and bind to and accumulate in the dorsal root ganglia (DRG) and peripheral axons [8, 14, 15]. In addition to neurotoxicity, this may cause neuroinflammation through activation of immune and immune-like glial cells, such as periganglionic satellite glial cells [16], followed by secretion of mediators that enhance neuronal excitability and generate pain hypersensitivity [17]. Indeed, recent studies have demonstrated the involvement of both innate and adaptive immune responses in CIPN. For example, treatment with PTX was associated with infiltration of macrophages and pro-inflammatory T cells into the DRG [18–20]. Furthermore, studies have reported sensitisation of DRG primary sensory neurons in PTX-induced peripheral neuropathy through induction of monocyte chemoattractant protein-1(MCP-1/CCL2) and its receptor CCR2, as well as toll-like receptor 4 (TLR-4) signalling in the DRG via MAP kinases and NFkB [21–23].

Neuroinflammation has also been shown to occur in the spinal cord during CIPN. Accumulating evidence suggests that CIPN is associated with activation of spinal astrocytes with no significant involvement of microglia [24, 25]. OXA treatment in rats caused mechanical hypersensitivity, which coincided with hyper-activation of astrocytes, upregulation of pro-inflammatory cytokines (tumour necrosis factor (TNF)-α and interleukin (IL)-1β) and downregulation of anti-inflammatory cytokines (IL-10 and IL-4), and was independent of T-cell infiltration in the spinal cord [26]. Inhibition of this astrocyte-associated neuroinflammatory response by A3 adenosine receptor agonists blocked the development of PTX-induced neuropathic pain [27]. Furthermore, astrocyte activation in CIPN has been linked to downregulation of glutamate aspartate transporter (GLAST) and glutamate transporter-1 (GLT-1), as well as an increase in astrocyte-specific gap junction protein connexin 43, which may contribute to neuropathic pain [25, 28].

In addition to inducing neuroinflammation through neurotoxicity, chemotherapy can also modulate the systemic immune response. For example, studies in humans and animal models have reported systemic T-reg cell impairment following treatment with PTX [29, 30]. This may favour an elevation of the pro-inflammatory response, however whether alterations in peripheral immunity directly contribute to CIPN pathology and clinical symptoms is yet to be determined.

Despite our increasing understanding of the key mechanisms that drive neuropathic pain in experimental CIPN, the interplay between the immune and nervous systems in CIPN remains unclear. In the current study, we investigated neuroimmune changes in an experimental mouse model of CIPN using clinically relevant doses of either PTX (commonly used to treat breast, ovarian and lung cancers) or OXA (commonly used to treat colorectal cancer); two frontline chemotherapeutics with different mechanisms that are known to cause CIPN. We demonstrate that mice administered with either PTX or OXA develop mechanical pain hypersensitivity and exhibit differential changes in peripheral immune responses and neuroinflammation in the PNS and the spinal cord at initial and peak stages of CIPN development.

## Materials and Methods

### Animals

C57BL/6J and DEpletion of REGulatory T cell (DEREG; on C57BL/6J background) male mice (Australian BioResources, Moss Vale, NSW, Australia) 8–12 weeks old were used. Animals were group housed in sterile, individually ventilated cages with chow and water *ad libitum*. A 12:12 hour light/dark cycle was maintained and housing was kept at a constant room temperature (RT) and humidity levels. All experiments were approved by the Animal Care and Ethics Committee of the University of New South Wales and were conducted in accordance with the guidelines issued by the International Association for the Study of Pain.

### Chemotherapy treatment

A stock solution of PTX (Tocris Bioscience; 15mg/ml) was prepared in Cremaphor (Sigma Aldrich) and ethanol in a 1:1 ratio and further diluted in 0.9% sterile saline to a final injected dose of 5mg/kg in 50μL/mouse. A stock solution of OXA (Sigma-Aldrich; 5 mg/ml) was prepared in 0.9% saline and further diluted with sterile saline at a 1:1 ratio to a final injected dose of 5mg/kg in 50μL/mouse. Mice were injected on alternate days (days 0, 2, 4, 6) intraperitoneally (i.p.) with a cumulative dose of 20mg/kg (equals 740mg/m^2^ in humans) distributed over 4 injection cycles. Control mice were injected i.p. with 50μL of 0.9% sterile saline over 4 alternating days to match the injection volume and cycle of the chemotherapy drugs.

### Behavioural tests

Tests of mechanical pain hypersensitivity were conducted before chemotherapy to give a baseline measurement and every 3 to 4 days following chemotherapy until a designated end-point. Prior to testing, mice were allowed 45 minutes to 1 hour to habituate in the testing apparatus. Both left and right hindpaws were tested. Mechanical allodynia was assessed by using the “up-and-down” method, as previously described by Dixon (1965) and Chaplan *et al* (1994) [31, 32]. Calibrated von Frey filaments (North Coast Medical, Gilroy, CA, USA; ranging from 0.07 grams to 4 grams) were applied to the mid-plantar surface of the hindpaw for 3 to 4 seconds. A positive response was recorded if the mouse showed withdrawal and guarding, “scratching” or “licking” of the stimulated paw. At least six bilateral measurements were taken for each animal. Data were calculated as the 50% withdrawal threshold in grams. Mechanical allodynia was defined as a significant reduction in paw withdrawal threshold (PWT) relative to baseline measurements. The experimenter was blinded to treatment during testing and analyses.

### Immunohistochemistry

CIPN and control mice were deeply anesthetised with 0.1 ml Letharbarb i.p. and transcardially perfused with 0.9% saline containing heparin, followed by 10% formalin (Sigma Aldrich) for fixation. Lumbar spinal cord (SC; L3 to L5 region), bilateral sciatic nerves and bilateral L3/L4 DRGs were dissected and postfixed overnight in 10% formalin. Tissue was stored in 30% sucrose solution (with 0.1% Sodium Azide) at 4°C and then embedded in OCT medium just prior to sectioning. Using a cryostat, the DRGs and sciatic nerves were sectioned longitudinally at 10μm thickness and the SC was sectioned transversely at 20μm thickness. All sections were mounted on superfrost plus slides (Thermo Fisher Scientific, Australia) and air-dried overnight at RT. The sections were fixed for 10 min at RT with 80% (v/v) absolute ethanol and washed twice in distilled water and once in phosphate buffered saline (PBS) containing 0.05% Tween-20 (PBS-T). All sections were blocked for 1 hr at 37°C with blocking solution (PBS containing 5% donkey serum, 0.2% Tween-20 and 0.3% Triton-X). Sections were then incubated for 1 hr at RT or overnight at 4°C with the following primary antibodies: mouse monoclonal GFAP for astrocytes (1:1000, Millipore Corporation, Canada); rabbit anti-IBA1 for microglia/macrophages (1:2000, Wako Pure chemical industries Ltd, Osaka Japan); rabbit anti-activating transcription factor-3 (ATF-3) for damaged neurons (1:400, Santa Cruz Biotechnology Inc, TX, USA); mouse monoclonal anti-neurofilament 200 (NF200) for large myelinated afferent cell bodies (1:1000, Sigma-Aldrich, Australia); goat anti-calcitonin gene related peptide (CGRP) for peptidergic unmyelinated afferent cell bodies (1:1000, Abcam, Australia); purified rat anti-mouse CD45R for B-cells (1:200, BD Biosciences, San Diego, CA, USA); rat anti-mouse CD3 for T cells (1:100, R&D systems, MN, USA); and rat monoclonal anti-neutrophil (7/4) for neutrophils (1:2500, Abcam, Sapphire Biosciences Pty Ltd, Cambridge UK) diluted in PBS containing 5% bovine serum albumin (BSA). Sections were washed four times with PBS-T for 10 min each and incubated with the appropriate secondary antibody: Alexa Fluor 488 donkey anti-mouse (1:1000; Life Technologies, Mulgrave, VIC, Australia); Alexa Fluor 488 or 546 donkey anti-rabbit (1:1000; Life Technologies, Mulgrave, VIC, Australia); Alexa Fluor 488 donkey anti-goat (1:1000, Mulgrave, VIC, Australia); Donkey anti-rat IgG conjugated with Cy2 (1:100, Jackson ImmunoResearch, West Grove PA, USA) diluted in PBS containing 5% BSA for 1 hr at RT. Sections were then washed four times with PBS-T for 10 min each. For P2ry12+ microglia, immunostaining was carried out as previously described [33]. Briefly, sections were immersed in 70% ethanol for 5 min, washed twice in distilled water, followed by incubation in PBS-T for 5 min at RT. Sections were blocked with a blocking solution (10% goat serum, 2% BSA, 1% glycine, 0.01% Triton-X in PBS) for 1hr at RT, and were then incubated with rabbit anti-P2ry12 primary antibody (1:500, provided by Dr. Oleg Butovsky) diluted in PBS containing 5% goat serum and 0.2% Triton-X overnight at 4°C. Sections were washed three times with PBS-T (5 min each) and incubated with a secondary antibody (Alexa Fluor 488 goat anti-rabbit; 1:800, Life Technologies, Mulgrave, VIC, Australia) diluted in blocking solution for 1 hr. Sections were then washed three times with PBS-T (5 min each). For isolectin B_4_ (IB4) (non-peptidergic unmyelinated afferent cell bodies) and ATF-3 double staining, sections were stained with ATF-3 (as described earlier). After incubation with secondary antibody, sections were washed twice with PBS-T (10 min each) and twice with PBS (10 min each), followed by a 2 hr incubation with IB4 diluted in PBS (1:100, Sigma-Aldrich, Australia). Sections were then washed four times with PBS for 10 min each. After immunostaining, prolong gold anti-fade reagent with 4', 6-diamidino-2-phenylindole (DAPI; Life Technologies, Mulgrave, VIC, Australia) was applied before slides were cover-slipped and stored at 4°C until viewing.

### Image acquisition and analysis

All images were viewed using an Olympus BX51 epifluorescence microscope, and images captured using an Olympus DP73 camera using CellStandard software (Olympus, Tokyo, Japan). Images were captured from 4 to 6 stained sections for each animal. The experimenter was blinded to the treatment groups during image analysis.

For immune cell (monocytes/macrophages, T-cells, B-cells, neutrophils) analysis, slides containing 4–6 sections of L3-L4 DRGs, sciatic nerve or L3-L5 spinal cord segment for each animal were stained for a given antibody and imaged at 20X magnification. Cells positive for both the antibody and DAPI were manually counted using NIH ImageJ software (NIH, Bethesda, USA), and expressed as a percentage of total area of the section in the field of view. Cell counts from 4–6 sections for sciatic nerves and 4–6 sections for each L3 and L4 DRGs per animal were averaged.

For characterisation of injured sensory neurons, slides containing 4–6 sections of L3-L4 DRGs for each animal were immuno-labelled with ATF-3 and IB4, CGRP or NF200, and imaged at 20X magnification. The number of neurons with cell body nuclei positively stained for ATF-3 were manually counted using Image J software and expressed as a percentage of all DRG cell bodies in the image field. In addition, the numbers of double-labelled ATF-3 and IB4, CGRP or NF200 neurons were manually counted and expressed as a percentage of total ATF-3 positive neurons. Cell counts from 4 to 6 sections were averaged for each L3 and L4 DRGs per animal.

For glial cell (astrocytes and microglia) analysis, L3-L5 spinal cord sections were stained with GFAP, IBA1 and P2ry12 antibodies, and images were taken at 40X magnification of the left and right ventral and dorsal horn regions, as previously described [34]. Image J software was used to calculate the percentage of total immunoreactivity within the spinal cord region following subtraction of background. Values for left and right dorsal or ventral horn were averaged for each spinal cord section and 4–6 sections were analysed for each animal.

### Flow cytometry

At designated end-points, mice were deeply anaesthetised. The spleen and inguinal lymph nodes were removed and stored in PBS on ice, and blood (0.4–0.5mL per mouse) was withdrawn from the left ventricle using a 0.5mL syringe with a 30G needle and collected into a tube containing 1mL anticoagulant citrate dextrose solution and stored on ice. Mice were then transcardially perfused with heparinised 0.9% saline solution and the L3-L4 DRGs and whole spinal cord were dissected and stored in PBS on ice.

Each tissue was processed separately to achieve single cell suspensions as follows. Five mL of red blood cell lysis buffer (Alfa Aesar, Lancashire, UK) was added to spleen and blood samples, which were then incubated for 5 min with gentle shaking. Samples were centrifuged at 600xg at 4°C for 5 min, before discarding the supernatant. Lymph node samples were crushed in a 1.5mL tube using a pestle, and passed through a 40μm cell strainer in 10mL PBS. Samples were then centrifuged at 600xg at 4°C before removing the supernatant. Nervous system tissue was coarsely chopped and 1mL of Accutase (Sigma-Aldrich, NSW, Australia) was added to each sample for a 30 min incubation at 37°C with 5% CO_2_. Following digestion, nervous system tissues were mechanically grinded through 70μm cell strainers in 10mL PBS. Samples were then centrifuged at 600xg at 4°C for 5 min and the supernatant was discarded.

All samples were resuspended in auto Magnetic-activated Cell Sorting (MACS) running buffer to a cell count of ~1 x 10^7^cells/mL. Cells undergoing cytokine staining were resuspended in RPMI-1640 media (Sigma-Aldrich) at 2 to 4 x 10^6^ cells/mL. Cell activation cocktail with Brefeldin A (Biolegend, San Diego, CA, USA) was added according to manufacturer’s instructions and incubated at 37°C with 5% CO2 for 4 hours. Activated cells were then resuspended in autoMACS running buffer to a concentration of ~1 x 10^7^ cells/mL. Cells in autoMACS running buffer (100μL) were added to separate tubes, and cell surface markers were stained for 30 min at 4°C with the following combinations of antibodies: anti-mouse CD45-APC (1:1000; eBioscience, San Diego, CA, USA), anti-mouse CD4-FITC (1:1000; eBioscience), anti-mouse CD8-PeCy7 (1:1000; eBioscience), anti-mouse CD25-APC (1:400; eBioscience), or suitable isotype controls. Cells were then washed three times in 1mL autoMACS running buffer (5 min, 600xg), before being resuspended in fixation/permeabilisation solution (eBioscience) and incubated overnight at 4°C. The following day, samples were washed twice with permeabilisation buffer (eBioscience), and intracellularly stained with rat anti-mouse/rat Foxp3-PE (1:200; eBioscience), anti-mouse IFN-γ-APC (1:100; Biolegend), anti-mouse IL-17α-PE (1:200; Biolegend), anti-mouse IL-4-PerCP/Cy5.5 (1:200; Biolegend), or suitable isotype control antibodies in permeabilisation buffer for 30 minutes at room temperature. Finally, samples were washed 3 times with permeabilisation buffer and re-suspended in 200μL autoMACS running buffer. Samples were then acquired using a BD FACS Canto II flow cytometer using BDFACS DIVA software (Becton Dickinson (BD) Biosciences, Franklins Lakes, NJ, USA), and analysed with FlowJo software (FlowJo, OR, USA). A minimum of 30,000 events were acquired for each sample.

### Cytokine assay

Mice were deeply anaesthetised and blood was extracted from the heart. Whole blood (>200 μl per mouse) was allowed to clot for at least 1 hour at RT before centrifugation and the serum supernatant was removed and stored at −80°C. Mice were then transcardially perfused with heparinised 0.9% saline solution and spinal cord and DRG tissue samples were rapidly dissected and snap frozen in liquid nitrogen. Protein was extracted from these tissues by bead homogenization in a Precellys homogenizer (Bertin Technologies) followed by treatment with Bio-Plex Cell Lysis Kit (Bio-rad, Hercules, CA, USA). Protein concentration was determined using a bicinchoninic acid protein assay kit (Bio-rad, Hercules, CA, USA) and 25μg of spinal cord and 10μg of DRG protein was used per animal. Serum samples were diluted 1:4 according to manufacturer’s instructions. All samples were measured using Bio-plex Pro Mouse Cytokine 23-Plex (Bio-rad Hercules, CA, USA) assay according to the manufacturer’s instructions. Data were collected and analysed using a MAGPIX^™^ Multiplex Reader (Merck Millipore, MA, USA), and results were converted to a pg/ml concentration for all of the cytokines tested. Individual values were subtracted from background, and sample concentrations that were lower than background or below detection limit were assigned a value of zero. Individual cytokines were excluded from analysis if the number of values assigned as zero equalled or exceeded 80% of total number of values.

### Regulatory T-cell depletion

T-reg cells were depleted in DEREG mice by administration of Diphtheria Toxin (DT) diluted in Dulbecco’s-PBS vehicle (Invitrogen). DT was injected i.p. at a concentration of 500ng/100μL on days 7, 12, 17 and 22 following OXA injection cycle (days 0, 2, 4 and 6). DEREG mice injected with Dulbecco’s PBS vehicle only following OXA injection cycle, and DEREG mice injected with 0.9% saline (days 0, 2, 4 and 6) and Dulbecco’s PBS vehicle were used as controls.

### Statistical analysis

All data (except for cytokine assay) are expressed as mean and standard error (mean±SEM). Statistical analyses and figures were generated using GraphPad Prism software version 6.03. Behavioural data were analysed using two-way repeated measures (RM) analysis of variance (ANOVA) followed by Bonferroni’s multiple comparisons test. Immunohistochemistry and flow cytometry data were analysed using one-way ANOVA followed by Bonferroni’s multiple comparisons test. Cytokine assay data deemed as not-normally distributed were expressed as median and interquartile range, and analysed non-parametrically using the Kruskal-Wallis test followed by Dunn’s multiple comparisons test. Data points were removed if they were identified as outliers by the Regression and OUTlier removal (ROUT) method (Q = 1%). A p-value less than 0.05 was considered statistically significant.

## Results

### Effect of chemotherapy on mechanical pain hypersensitivity in male C57BL/6J mice

**Fig 1:**
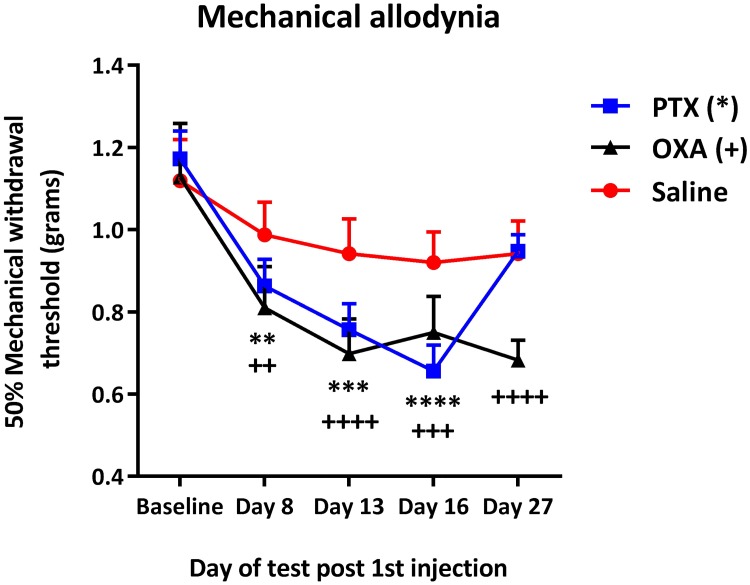
Effect of chemotherapy on mechanical sensitivity in C56BL/6J male mice. Time course of mechanical paw withdrawal threshold (PWT; in grams) of male C56BL/6J mice treated with paclitaxel (PTX), oxaliplatin (OXA) or saline (control). Measurements were taken from baseline before chemotherapy until day 27, with chemotherapy injections administered at days 0, 2, 4 and 6. Compared to baseline thresholds, significant decrease in PWT was seen on days 8, 13, and 16 in the PTX-treated group, and on days 8, 13, 16 and 27 in the OXA-treated group. **(P<0.01); ***(P<0.001); ****(P<0.0001) indicate significant difference between baseline and post-chemotherapy time points in the PTX group. ^++^(P<0.01); ^+++^(P<0.001); ^++++^(P<0.0001) indicate significant difference between baseline and post-chemotherapy time points in the OXA group. PTX (n = 16), OXA (n = 16), Saline (n = 15). RM two-way ANOVA followed by Bonferroni’s multiple comparisons test. Data are expressed as mean±SEM.

In line with previous studies in rodents [35, 36], we found that male C57BL/6J mice treated with PTX or OXA developed long-term hind paw mechanical allodynia (Fig 1), as demonstrated by a significant reduction in paw withdrawal thresholds on days 8, 13 and 16 post 1^st^ injection. Saline-treated mice did not develop mechanical pain hypersensitivity at any time point over the course of the experiment.

### Effect of chemotherapy on systemic immune responses

**Fig 2:**
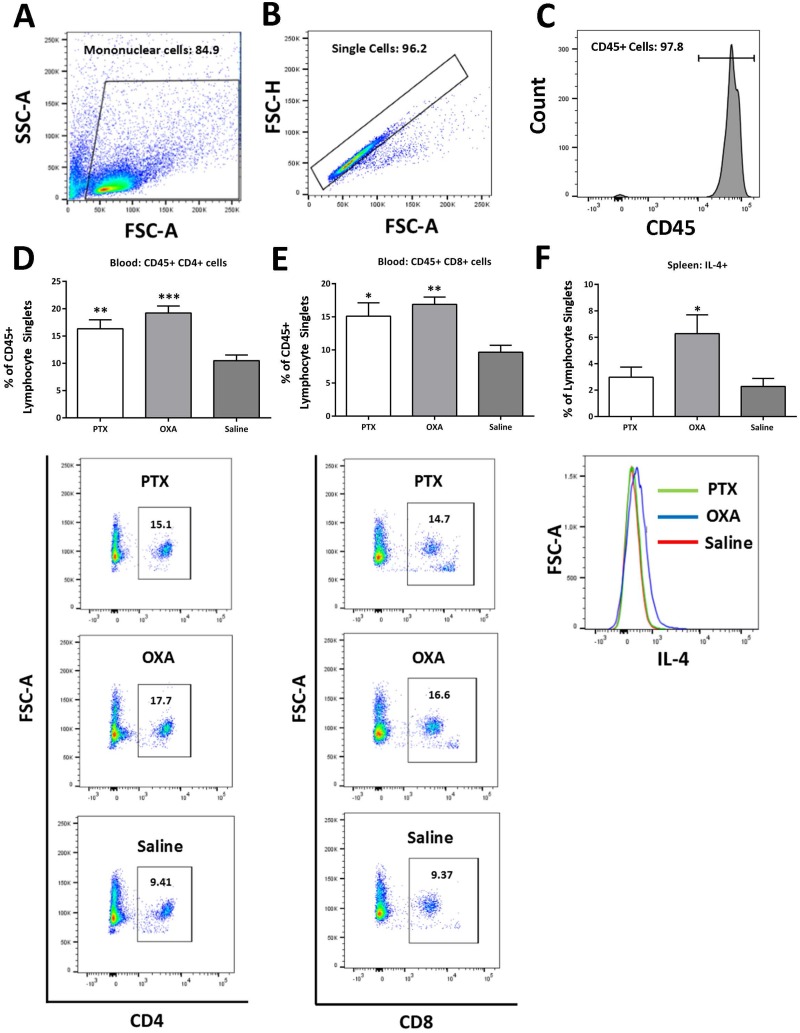
Systemic inflammatory changes in the blood and spleen. Flow cytometry of lymphocytes to characterise inflammatory changes in the blood and spleen was carried out on day 7 post-1^st^ paclitaxel (PTX), oxaliplatin (OXA) or saline (control) injection. Mononuclear cells were first gated (A), followed by consecutive gating of singlets (B). For blood, singlets were further gated for CD45+ lymphocytes (C). Representative flow cytometric plots and column graphs of CD45+CD4+ (D) and CD45+CD8+ (E) T-cell populations in the blood (expressed as a percentage of CD45+ lymphocyte singlets), and total IL-4+ lymphocytes (F) in the spleen (expressed as a percentage of lymphocyte singlets). Significant increase in CD45+CD4+ and CD45+CD8+ T-cell populations in the blood of PTX- and OXA- treated mice (n = 10), and IL-4 positive lymphocytes in the spleens of OXA-treated mice (n = 7–9) compared to saline controls were found. *P<0.05; **P<0.01; ***P<0.001 indicate significant difference between PTX/OXA and saline controls. One-way ANOVA followed by Bonferroni's multiple comparison’s test. Data are expressed as mean±SEM.

Chemotherapy-induced neuropathic pain may be mediated by activation of peripheral immunity and subsequent stimulation of secondary neuroimmune pathways [37–40]. Therefore, to study the effects of PTX and OXA on peripheral immune responses, we performed flow cytometry to quantify immune cells and cytokines in the inguinal lymph nodes, spleen and blood at the initial (day 7 post 1^st^ injection) and developing (day 13 post 1^st^ injection) stages of PTX- and OXA-induced neuropathic pain. Mononuclear cells were first gated, followed by selection of live singlets (Fig 2A and 2B) from which we measured lymphocyte subsets and intracellular cytokines as percentages of lymphocyte singlets.

In the blood, we gated live singlet cells for CD45+ lymphocytes (Fig 2C), followed by subsequent gating for CD4 and CD8 T-cell subsets. We observed a significant increase in CD45+CD4+ (Fig 2D) and CD45+CD8+ (Fig 2E) T-cells on day 7 (a day after the last injection) in PTX- and OXA-treated mice compared to saline controls. In the inguinal lymph nodes, CD4+ T-cell levels were unchanged across the treatment groups, as were the IFN-γ, IL-17α and IL-4 positive lymphocytes (S1A–S1D Fig). In the spleen, no significant changes were observed (S2A–S2C Fig), except for a significant increase in IL-4+ splenocytes in OXA-treated mice compared to saline controls on day 7 (Fig 2F).

**Fig 3:**
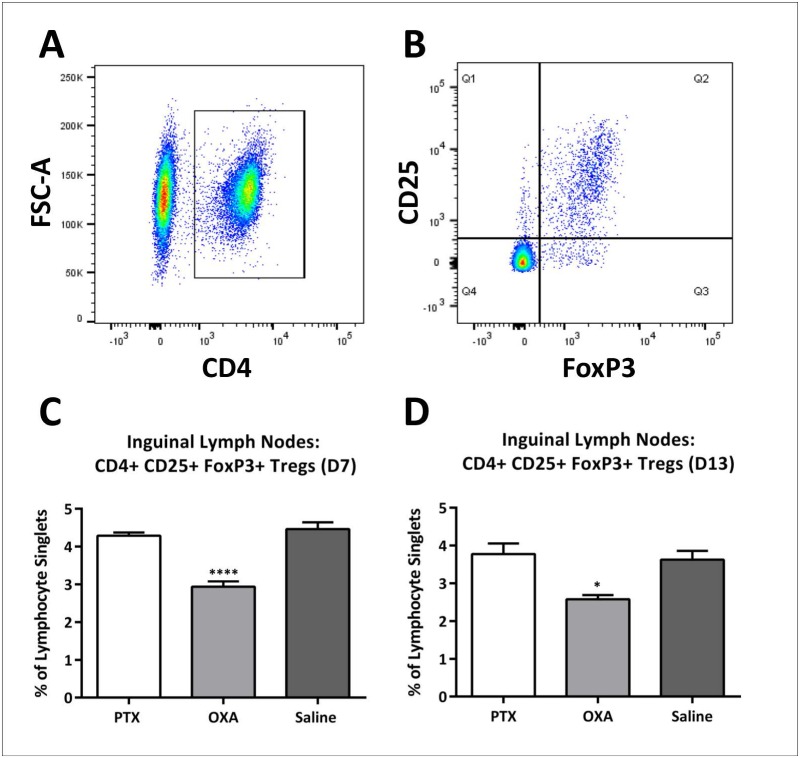
Changes in regulatory T-cell population in inguinal lymph nodes following chemotherapy. Flow cytometry of lymphocytes to characterise regulatory T-(T-reg)-cell changes in inguinal lymph nodes on days 7 and 13 post-1^st^ injection of paclitaxel (PTX), oxaliplatin (OXA) or saline (control) was carried out. CD4+ cells were first gated from lymphocyte singlets (A), followed by consecutive gating of CD25+FoxP3+ cells (B). Column graphs of CD4+CD25+FoxP3+ T-reg cell populations in the lymph nodes on day 7 (C) and day 13 (D) are expressed as a percentage of lymphocyte singlets. A significant decrease in T-reg cells was seen in OXA-treated mice on both day 7 (n = 6, ****P<0.0001) and day 13 (n = 4, *P<0.05) compared to saline control mice. One-way ANOVA followed by Bonferroni's multiple comparison’s test. Data are expressed as mean±SEM.

In addition, we measured T-reg cell levels by gating lymphocyte singlets for CD4, followed by consecutive gating for CD25 and FoxP3 (Fig 3A and 3B). We found a marked reduction in CD4+CD25+FoxP3+ T-reg cells in the lymph nodes of OXA-treated mice compared to saline controls on day 7 (Fig 3C). However, there were no T-reg cell changes in the spleens of chemotherapy-treated mice (S2D Fig).

On day 13 (a week after the last injection), the increase in CD4+ and CD8+ T-cells in the blood detected on day 7 normalised to saline control levels (S3A and S3B Fig). Furthermore, analysis of the cytokine profile in the serum of chemotherapy-treated mice on day 13 using a cytokine assay showed no significant changes in serum cytokine levels compared to saline controls (S1 Table). Similar to day 7, CD4+ T-cell levels in the inguinal lymph nodes were unaffected by chemotherapy on day 13, as were the levels of total IFN-γ, IL-17α and IL-4 positive lymphocytes (S1E–S1H Fig). There were no significant changes in CD4+ T-cell and cytokine levels in the spleen on day 13, with the initial day 7 increase in IL-4 cytokine in the OXA-treated group normalising to saline control levels (S2E–S2H Fig). However, OXA-induced reduction in T-reg cells in the lymph nodes (day 7) was sustained on day 13 (Fig 3D).

### Effect of regulatory T-cell depletion on chemotherapy-induced mechanical allodynia

**Fig 4:**
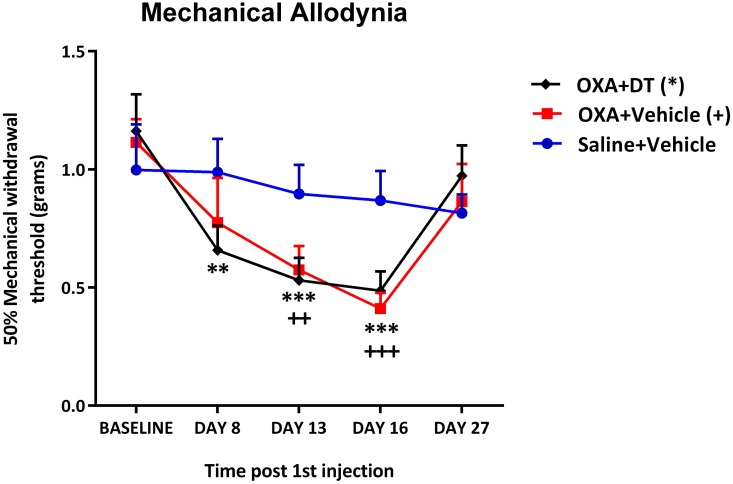
Effect of regulatory T-cell repletion on oxaliplatin-induced mechanical allodynia in transgenic DEREG mice. Time course of paw withdrawal threshold (PWT, in grams) of male transgenic DEREG mice administered with saline (days 0, 2, 4, 6) and vehicle (days 7, 12, 17, 22), and oxaliplatin (OXA; days 0, 2, 4 and 6) with either Diphtheria Toxin (DT) or vehicle (days 7, 12, 17 and 22). Measurements were taken from baseline until day 27 post-1^st^ injection. Compared to baseline thresholds, significant decrease in PWT was seen on days 8, 13, and 16 in the OXA+DT group, and on days 13 and 16 in the OXA+Vehicle group. There was no significant reduction in PWT in the Saline+Vehicle control group. **(P<0.01); ***(P<0.001) indicate significant difference between baseline and post-chemotherapy time points in the OXA+DT group. ^++^(P<0.01); ^+++^(P<0.001); indicate significant difference between baseline and post-chemotherapy time points in the OXA+Vehicle group. No significant difference was seen in PWT at any time point between OXA+DT and OXA+Vehicle groups. RM two-way ANOVA followed by Bonferroni’s multiple comparisons test, n = 9 (OXA+DT), n = 8 (OXA+Vehicle), n = 6 (Saline+Vehicle). Data are expressed as mean±SEM.

Since OXA treatment induced a significant reduction of T-reg cells in the lymph nodes, we next examined the possible role of T-reg cells in mediating mechanical allodynia by conditionally ablating T-reg cells in transgenic DEREG mice. DEREG mice express a Diphtheria Toxin Receptor-enhanced green fluorescent protein (eGFP) under the control of the FoxP3 locus [41]. Since Foxp3 is regarded as the most specific T-reg cell marker [42], DEREG mice allow for highly specific depletion of T-reg cells *in vivo* with DT. Here we compared mechanical pain hypersensitivity between T-reg-depleted and non-depleted DEREG mice following OXA treatment. Both groups of DEREG mice treated with OXA developed mechanical pain hypersensitivity, as demonstrated by a significant decrease in paw withdrawal thresholds on days 13 and 16 post 1^st^ injection. However, we found no change in paw withdrawal thresholds between T-reg-depleted and non-depleted OXA-treated DEREG mice; Compared to mice administered with vehicle, repeated treatment with DT did not alter OXA-induced mechanical pain hypersensitivity over the course of behavioural testing (Fig 4).

### Effect of chemotherapy on the peripheral and central nervous system

Since mechanical pain hypersensitivity was established by day 13, we next examined glial and immune cell changes in the peripheral (sciatic nerve and DRG) and central (spinal cord) nervous systems of PTX- and OXA-treated mice on day 13 post 1^st^ injection.

#### Peripheral nervous system

**Fig 5:**
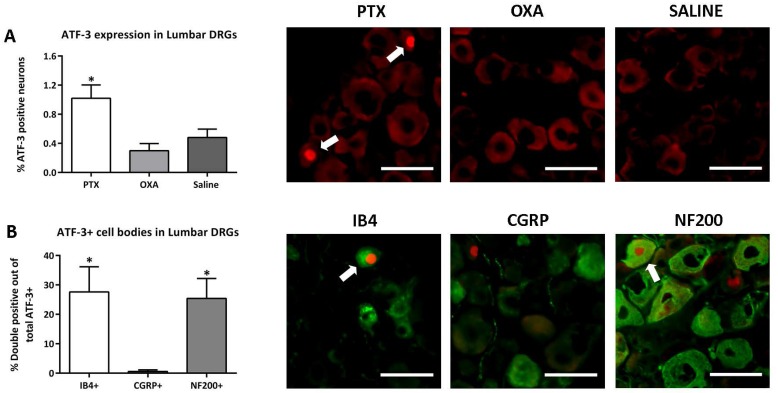
Chemotherapy-induced neuronal injury in the lumbar DRG. ATF-3, IB4, CGRP and NF200 immunohistochemistry in L3-L4 DRG was carried out on day 13 post-1^st^ injection of paclitaxel (PTX), oxaliplatin (OXA) or saline (control). (A) Column graph and representative images depicting neuronal ATF-3 expression in the DRG, which was significantly higher in the PTX group compared to saline control group (*P<0.05). Scale bar = 50 μm. (B) Column graph and representative images showing double immunolabelling of ATF-3 with IB4, CGRP and NF200 in the DRG of PTX-treated mice. ATF-3 was predominantly expressed in IB4+ and NF200+ DRG neurons in the PTX group (*P<0.05). Scale bar = 50μm. n = 3–4, one-way ANOVA followed by Bonferroni's multiple comparison’s test. Data are expressed as mean±SEM.

To test the effects of the chemotherapeutic drugs on neuronal injury, we carried out immunohistochemistry for ATF-3, a marker of cells with damaged peripheral axons, in the L3-L4 DRGs. As compared to saline-treated mice, there was a small but significant increase in sensory neurons expressing ATF-3 in PTX-treated mice, but not in OXA-treated mice (Fig 5A). Further characterisation showed that ATF-3 was co-localised with IB4+ and NF200+ labelled DRG neurons in the PTX-treated mice (Fig 5B).

**Fig 6:**
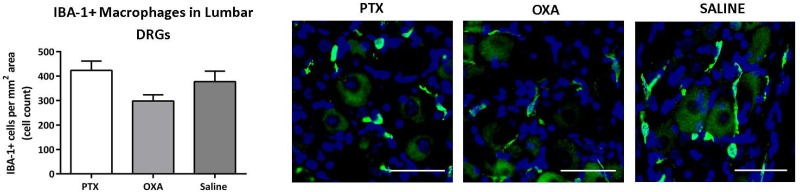
Chemotherapy-induced immune cell changes in the lumbar DRG. IBA-1 immunohistochemistry in L3-L4 DRG was carried out on day 13 post-1^st^ injection of paclitaxel (PTX), oxaliplatin (OXA) or saline (control). Column graph and representative images of IBA-1/DAPI positive macrophages in the DRG showing no significant differences across treatment groups. Scale bar = 50μm. n = 4, one-way ANOVA followed by Bonferroni's multiple comparison’s test. Data are expressed as mean±SEM.

Immune cell infiltration and altered cytokine expression in the peripheral nerve and DRG have been implicated in animal models of neuropathic pain [19, 20, 34, 43]. Therefore, the DRGs were further examined for chemotherapy-induced immune cell infiltration (using immunohistochemistry and flow cytometry) and cytokine changes (using Bio-plex cytokine assay). No significant changes in IBA-1 expressing macrophages/monocytes (Fig 6), CD45+CD4+ and CD45+CD8+ T-cells (S4A and S4B Fig; using similar gating as in Fig 2A–2C), and CD3+ T-cells, B-cells and neutrophils (data not shown) were detected in PTX- and OXA-treated groups. However, cytokine profiling of the DRG showed several significant changes relative to saline controls with a 0.55-fold decrease in chemokine (C-C motif) ligand (CCL)4 chemokine in OXA-treated animals, a 1.4-fold increase in CCL3 and a 2.1-fold increase in CCL2 in PTX-treated animals (Table 1). As in the DRG, the sciatic nerve did not show any significant changes in levels of infiltrating macrophages/monocytes, CD3+ T-cells, B-cells and neutrophils (data not shown).

**Table 1:** Cytokine and chemokine changes in the DRGs following chemotherapy.

Cytokine and Chemokine	PTX-median (interquartile range)	OXA-median (interquartile range)	Saline-median (interquartile range)
**IL-1β**	59.5 (57.6–68.9)	63.3 (38.5–63.3)	51.86 (46.1–61.4)
**TNF-α**	23.3 (20.7–34.1)	34.0 (15.1–35.8)	16.6 (13.5–25.1)
**IFN-γ**	1.4 (1.2–1.6)	1.1 (0.9–3.0)	1.0 (0.8–1.5)
**IL-9**	50.5 (41.6–66.7)	26.8 (8.6–44.0)	11.2 (1.5–45.6)
**IL-2**	12.8 (11.8–13.9)	10.5 (10.4–10.9)	10.1 (6.2–14.2)
**IL-13**	53.5 (45.8–56.1)	40.7 (35.8–48.4)	45.8 (35.8–51.0)
**IL-6**	6.9 (5.9–7.9)	4.8 (3.5–5.6)	4.8 (3.4–7.0)
**IL-1α**	15.0 (14.7–16.5)	13.5 (10.9–13.6)	12.4 (8.6–15.3)
**CCL5**	1.8 (1.2–2.1)	0.7 (0.3–1.1)	1.0 (0.6–1.8)
**IL-10**	5.7 (5.0–7.2)	4.2 (3.9–6.1)	3.5 (3.5–6.1)
**CCL11**	86.4 (77.1–97.4)	72.3 (57–79.5)	77.1 (67.3–92.8)
**CCL4**	1.3 (1.3–1.3)	1.3 (0.9–2.4) *	2.8 (2.1–3.6)
**CCL3**	1.5 (0.7–2.0) *	0 (0–1.5)	0 (0–0)
**IL-12p70**	6.2 (5.8–8.5)	5.7 (4.3–8.3)	4.6 (3.0–6.6)
**IL-12p40**	0.8 (0.8–1.0)	0.7 (0–0.9)	0.6 (0.5–0.8)
**CCL2**	11.5 (11.5–11.5) *	7.1 (3.0–7.1)	3.0 (3.0–9.3)
**GM-CSF**	30.5 (28.6–36.0)	18.7 (14.4–28.6)	18.7 (14.4–30.3)
**IL-17α**	0 (0–0)	1.9 (0–2.1)	0 (0–0)

Bio-plex analysis of cytokine and chemokine profile was carried out in L3-L4 DRGs on day 13 post-1^st^ injection of paclitaxel (PTX), oxaliplatin (OXA) or saline. Table showing median and interquartile range of cytokine/chemokine concentrations (pg/ml) in the DRGs (n = 5);

* P<0.05 indicates significant difference between PTX-/OXA- and Saline-treatment groups; Kruskal Wallis test followed by Dunn’s multiple comparisons test.

#### Central nervous system

**Fig 7:**
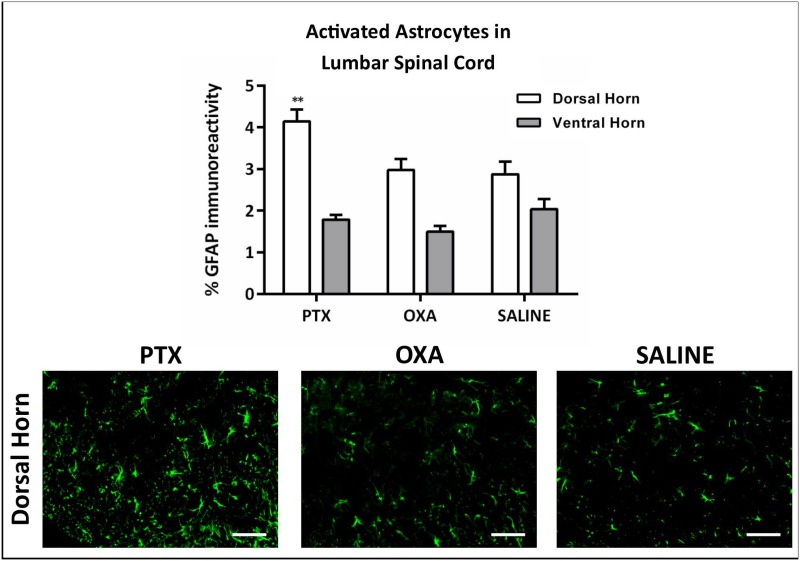
Chemotherapy-induced astrocyte activation in the spinal cord dorsal horn. GFAP immunohistochemistry in L3-L5 spinal cord was carried out on day 13 post 1^st^ injection of paclitaxel (PTX), oxaliplatin (OXA) or saline (control). Column graph showing the percentage of GFAP immunoreactivity in spinal cord dorsal and ventral horns, and representative images depicting GFAP immunoreactivity in the dorsal horn. GFAP immunoreactivity was significantly higher in the dorsal horn of PTX-treated mice compared with saline controls (**P<0.01), with no significant changes in the ventral horn. Scale bar = 50μm. n = 4, one-way ANOVA followed by Bonferroni's multiple comparison’s test. Data are expressed as mean±SEM.

**Fig 8:**
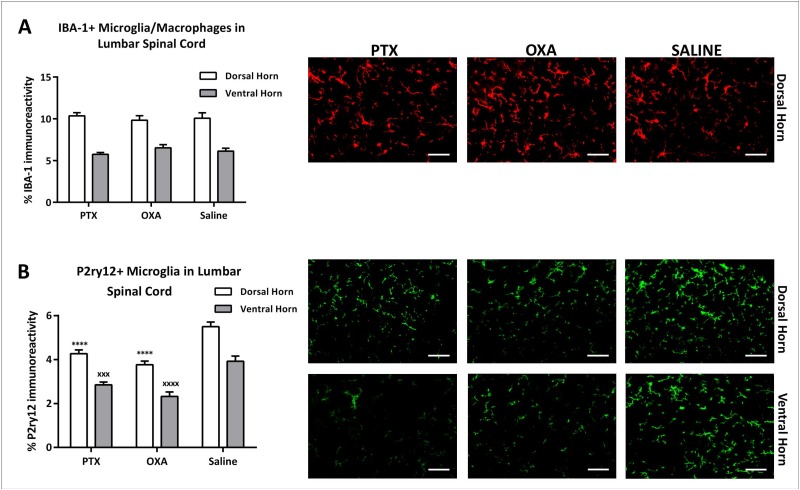
Chemotherapy-induced changes in microglia in the spinal cord. IBA-1 and P2ry12 immunohistochemistry in L3-L5 spinal cord segment was carried out on day 13 post-1^st^ injection of paclitaxel (PTX), oxaliplatin (OXA) or saline (control). (A) Column graph showing the percentage of IBA-1 immunoreactivity in the spinal cord dorsal and ventral horns, and representative images depicting IBA-1 immunoreactivity in the dorsal horn. No measurable changes in PTX (n = 4) or OXA (n = 3) group compared with saline (n = 4) were detected. Scale bar = 50 μm. (B) Column graph and representative images showing P2ry12 immunoreactivity in spinal cord dorsal and ventral horns, which was significantly lower in the PTX (n = 4) and OXA (n = 3) group, compared with saline controls (n = 3). Scale bar = 50 μm. ****P<0.0001 represents significant difference between PTX/OXA and saline in the dorsal horn. ^xxx^P<0.001, ^xxxx^P<0.0001 represent significant difference between PTX/OXA and saline in the ventral horn. One-way ANOVA followed by Bonferroni's multiple comparison’s test. Data are expressed as mean±SEM.

Studies have demonstrated marked changes in glial cell activation in the spinal cord of animals with CIPN [24, 28]. We therefore investigated immune-like glial cell changes in the lumbar spinal cord (L3-L5) of PTX- and OXA-treated animals on day 13. There was a significant increase in GFAP immunoreactivity in the spinal cord dorsal horn, but not ventral horn, of PTX-treated mice compared to saline controls, indicating astrocyte activation (Fig 7). However, no significant changes were observed in GFAP immunoreactivity in the dorsal or ventral horns of the spinal cord in OXA-treated mice compared to control mice. In addition, there were no significant changes in spinal cord microglia/macrophages (IBA-1 immunoreactivity) in the dorsal or ventral horn of CIPN animals compared to controls (Fig 8A). However, we noted a significant reduction in P2ry12+ homeostatic microglia in the spinal cord dorsal and ventral horns of PTX- and OXA-treated animals compared to controls (Fig 8B).

The spinal cord was further examined for immune cell infiltration and cytokine changes on day 13. Flow cytometric analysis of the spinal cord revealed no significant changes in CD45+CD4+ and CD45+CD8+ T-cell populations (S4C and S4D Fig; using similar gating as in Fig 2A–2C) and immunohistochemical analysis revealed no T cell/B cell infiltration into the spinal cord (data not shown). However, cytokine profiling of the spinal cord showed a significant 1.2-fold increase in TNF-α; 1.2-fold increase in IFN-γ; 1.2-fold increase in CCL11; 1.4-fold increase in CCL4; 1.2-fold increase in CCL3; 1.2-fold increase in IL-12p70; and 1.2-fold increase in GM-CSF in PTX-treated mice compared to control, with no significant changes detected in OXA-treated mice (Table 2).

**Table 2:** Cytokine and chemokine changes in the spinal cord following chemotherapy.

Cytokine and Chemokine	PTX -median (interquartile range)	OXA- median (interquartile range)	Saline- median (interquartile range)
**IL-3**	4.5 (4.1–4.7)	3.8 (3.5–4.1)	4.0 (3.8–4.1)
**IL-1β**	107.6 (72.6–113.0)	89.4 (85.7–97.6)	91.1 (74.6–110.3)
**TNF-α**	145.5 (141.2–162.9) *	132.5 (128.2–143.3)	126.1 (120.7–141.2)
**IFN-γ**	10.3 (9.7–11.3) *	9.5 (9.0–9.7)	8.9 (7.9–9.6)
**IL-2**	14.8 (13.4–16.4)	11.6 (9.6–13.3)	12.9 (10.4–14.8)
**IL-13**	173.4 (157.4–220.8)	141.4 (138.7–162.7)	165.4 (142.7–187.9)
**IL-6**	6.9 (4.8–8.6)	5.5 (4.9–7.2)	7.0 (5.4–7.3)
**IL-1α**	16.7 (13.1–18.0)	14.5 (10.0–15.5)	14.5 (11.0–16.1)
**CCL5**	4.1 (3.6–4.7)	3.4 (2.9–3.8)	3.7 (3.0–4.0)
**IL-10**	19.3 (18.5–22.5)	16.8 (15.2–18.1)	19.3 (17.8–20.7)
**IL-17α**	18.3 (17.9–18.5)	18.1 (16.8–18.6)	16.8 (15.7–17.93)
**CCL11**	193.7 (189.9–201.6) *	181.6 (165.6–190.6)	170.4 (155.5–181.6)
**CCL4**	17.2 (16.4–18.7) *	16.1 (14.3–17.5)	13.2 (10.3–14.3)
**CCL3**	9.0 (8.7–9.3) **	7.9 (7.5–8.2)	7.3 (6.7–7.9)
**IL-12p70**	33.3 (32.4–39.3) *	31.9 (27.1–32.9)	30.8 (27.9–31.2)
**IL-12p40**	3.4 (3.2–3.8)	3.0 (2.9–3.0)	3.1 (2.9–3.2)
**CCL2**	49.5 (47.6–57.3)	45.6 (45.6–50.5)	45.5 (41.5–49.5)
**CXCL1**	2.6 (2.4–4.4)	2.3 (2.0–2.4)	2.6 (1.9–3.6)
**GM-CSF**	82.1 (81.5–85.9) *	71.6 (70.2–75.6)	72.9 (67.4–76.3)

Bio-plex analysis of cytokine and chemokine profile was carried out in the spinal cord on day 13 post-1^st^ injection of paclitaxel (PTX), oxaliplatin (OXA) or saline. Table showing median and interquartile range of cytokine/chemokine concentrations (pg/ml) in in the spinal cord (n = 5);

* P<0.05,

** P<0.01 indicates significant difference between PTX- and Saline-treatment groups; Kruskal Wallis test followed by Dunn’s multiple comparisons test.

## Discussion

In the present study, we demonstrated that the chemotherapeutic drugs PTX and OXA induced pain hypersensitivity in male C57BL/6J mice, however, the immune responses and phenotypic changes in the PNS and the spinal cord differed considerably between the two drugs. In the periphery, both PTX and OXA significantly increased circulating CD4+ and CD8+ T-cells, while only OXA increased splenocyte IL-4 levels (day 7) and reduced T-reg numbers in the inguinal lymph nodes (days 7 and 13). In the DRG, we observed increased ATF-3 expression in small IB4+ and large NF200+ neurons and upregulated CCL2 and CCL3 chemokine levels in PTX-treated animals (day 13). In the spinal cord, PTX treatment was associated with significant astrocyte activation, and both PTX and OXA induced a significant reduction in P2ry12+ microglia, with no measurable changes in IBA-1-positive microglia/macrophages. In addition, PTX treatment also induced significant upregulation of several cytokines and chemokines in the spinal cord. Interestingly, we observed no infiltration of immune cells into the nervous system following PTX and OXA treatment, suggesting that neuroinflammatory changes are restricted to resident immune/glial cells in CIPN-affected animals. Supporting this view, depletion of systemic T-reg cells had no effects on pain hypersensitivity in OXA-treated mice. These findings indicate that PTX and OXA exert differential effects on the immune and nervous systems, presumably acting through distinct mechanisms in CIPN.

### Systemic inflammatory changes after chemotherapy

Few studies have investigated peripheral immune responses in experimental CIPN models [19, 21]. Here, we performed a comprehensive immunological characterisation in PTX- and OXA-treated mice and demonstrated marked changes in peripheral immunity following chemotherapy that corresponded to the initial stage of pain hypersensitivity. However, a majority of these changes normalised to control levels as the neuropathic pain behaviour progressed.

Although chemotherapy has been commonly reputed as immunosuppressive, accumulating evidence indicates that chemotherapy may modulate the immune environment to strengthen subsequent immunotherapy or cancer vaccine approaches [40, 44]. Indeed, we found a temporary selective increase in CD4+ and CD8+ lymphocytes following PTX and OXA treatment, as well as a significant increase in the percentage of IL-4+ splenocytes (day 7) and a reduction in lymph node T-reg cells at both initial and peak pain hypersensitivity (day 7 and 13, respectively) following OXA treatment. Whether these changes play a significant role in the development of CIPN is unknown.

T-reg cells are immunosuppressive cells that suppress autoreactive and inflammatory T-cells to maintain immune homeostasis [45]. Therefore, their elimination is expected to promote a systemic pro-inflammatory response [46] that may assist the process of inhibition of cancer cell proliferation. However, the significance of T-reg suppression and associated systemic inflammation in CIPN is unclear. Recent studies in humans and animal models have reported PTX-induced selective reduction in T-reg cells in the periphery and have proposed mechanisms such as upregulation of cell death receptor Fas (CD95) expression [29] or Bcl-2/Bax mediated apoptosis [47]. In contrast to these studies, we did not find any selective T-reg cell impairment in the periphery in PTX-treated mice. However, we observed OXA-induced T-reg cell impairment in the inguinal lymph nodes. To the best of our knowledge, no other study has reported downregulation of T-reg cells in OXA-induced peripheral neuropathy.

Previous studies from our group have shown that T-reg cells play an important role in neuropathic pain. Animals treated with the T-reg cell expanding CD28 superagonist showed significantly reduced pain hypersensitivity following nerve injury, while depletion of T-reg cells exacerbated mechanical allodynia [46, 48]. Studies in CIPN that have altered T-reg cells and associated cytokine activity using adoptive transfer of T-reg cells [19], IL-10 gene therapy [49] or administration of T-reg cell enhancing bee venom derived Phospholipase A_2_ [20] found such manipulations to decrease neuropathic pain severity. Hence, the potential significance of OXA-induced T-reg cell impairment on pain hypersensitivity was investigated by inducing systemic T-reg cell depletion in OXA-treated DEREG mice using serial administration of DT. Interestingly, and in contrast to our expectations, we found that systemic depletion of T-reg cells did not exacerbate OXA-induced mechanical pain hypersensitivity. These results suggest that changes in systemic T-reg cells may not contribute to neuropathic pain behaviour in OXA-induced peripheral neuropathy.

### Peripheral nervous system changes after chemotherapy

We observed phenotypic changes in the lumbar DRG of PTX-treated mice. In particular, PTX treatment caused an upregulation of ATF-3 in the DRG sensory neurons on day 13. Neuronal ATF-3 expression is reflective of peripheral sensory nerve damage [50], and has been previously reported in other CIPN models [51, 52]. Double immunolabelling showed that ATF-3 was predominantly expressed in IB4+ non-peptidergic unmyelinated C-fibre and NF200+ large myelinated Aβ fibre neurons in the DRG of PTX-treated mice. This suggests that PTX caused small and large fibre neuropathy in our animal model, in line with previous animal and clinical studies [14, 18, 53]. This may point to a distinct PTX-specific mechanism that causes sensory nerve damage and may subsequently contribute to neuropathic pain. It should be noted that previous studies in rodents have reported increased ATF-3 expression in the DRG following OXA treatment [54, 55] suggesting that differences in species, dosages, and treatment cycles may influence ATF-3 expression in the DRG. The reason ATF-3 expression was observed in our PTX-treated mice, but not in our OXA-treated mice is unknown. However, higher cumulative doses of OXA can lead to platinum accumulation in the DRG [15, 56] and may be required for ATF-3 induction in sensory neurons. Alternatively, neuronal injury in OXA-treated mice may occur in later stages than in PTX-treated mice. Thus, further studies investigating later time points would be useful in elucidating the temporal changes induced by each of these drugs.

There is some evidence supporting the role of immune cell infiltration (mainly macrophages) and pro-inflammatory cytokine activity in the PNS in CIPN [19, 20, 57]. In our model of CIPN, we found no infiltration of IBA1+ monocytes/macrophages, or CD4+/CD8+ T-cells, B-cells or neutrophils in the DRG or sciatic nerve on day 13 following PTX or OXA treatment. Due to the susceptibility of the DRG to neurotoxicity, we further analysed cytokine and chemokine levels on day 13. We found an increase in CCL2 and CCL3 chemokines in PTX-treated animals and a decrease in CCL4 chemokine in OXA-treated animals, compared to saline controls. CCLs are key chemokines that regulate migration and infiltration of monocytes/macrophages, as well as other immune cells [58–60]. Chemokine activity has been demonstrated in other models of neuropathic pain where immune cell migration and infiltration is a pathological hallmark [11]. However, the absence of leukocyte infiltration or resident macrophage activation in CIPN suggests that changes in the levels of chemokines in the DRG may have another source, such as activated periganglionic satellite glial cells [61, 62]. Our finding of increased CCL2 in the DRG of PTX-treated mice is in line with a recent study showing that PTX-induced CIPN is associated with the induction of CCL2 and its receptor CCR2 [21]. Zhang et al. further demonstrated that activation of paracrine CCL2/CCR2 signalling between DRG neurons plays a crucial role in the development of PTX-induced neuropathy [21].

The importance of CCL3 and CCL4 chemokine activity in the development and maintenance of neuropathic pain has been previously identified in several animal models of central and peripheral neuropathies [34, 63–65]. In CIPN, PTX treatment corresponded to an increase in CCL3 in rat spinal cord dorsal horn [66], which supports our findings. However, the significance of changes in CCL3 and CCL4 levels in the DRG is unclear. In addition, whether chemokine changes on day 13 are associated with resident immune cell activation or immune cell infiltration at an earlier or later time point remain unclear.

Our data suggest that PTX interacts with the PNS without a paralleling systemic immune activation, and induces pathological changes characterised by neuronal injury and changes in cytokine profile that may correspond to mechanical pain hypersensitivity. Even though we did not detect OXA-induced changes in the PNS using our markers, we cannot eliminate the possibility of interactions through other mechanisms, or at different time points.

### Central nervous system changes after chemotherapy

Chemotherapy-induced changes in the central nervous system (CNS) have been implicated in CIPN. In our animal model of PTX- and OXA-induced CIPN, we examined immune and immune-like glial cell changes in the spinal cord on day 13 post-1^st^ injection. Flow cytometric analysis of T-cell subsets in the spinal cord showed no changes in CD4+ and CD8+ T-cell populations and immunohistochemistry showed no presence of T cells. These findings support a previous study by Janes et al., who found that T-cell infiltration was absent in the spinal cord following treatment with OXA [26]. Therefore, the absence of an adaptive immune response in the spinal cord suggests that central mechanisms of CIPN are independent of systemic immune cell infiltration.

In agreement with previous studies, we found an increase in astrocyte, but not microglia, activation in the spinal cord following PTX treatment [28]. However, our model did not show astrocyte activation following OXA treatment, which is at odds with studies that have reported OXA-induced spinal astrocyte activation [24, 25]. Again, this points to a unique mechanism of PTX-induced CIPN that may be centrally mediated by astrocyte activation. Indeed, previous studies have shown downregulation of GLAST and GLT-1 in astrocytes [28], which upon selective inhibition can induce spontaneous pain [67] and augment post-synaptic glutamate receptor activity in the dorsal horn of the spinal cord [68]. The absence of spinal astrocyte activation in OXA-treated mice suggests that unlike PTX, astrocyte activation may be a contributing factor but not a critical phenotypic change for the development of pain hypersensitivity in rodents treated with OXA.

Interestingly, we also detected a significant downregulation of resident P2ry12 immunoreactive microglia in the dorsal and ventral horns of the spinal cord on day 13 in both PTX- and OXA-treated mice. A recent study showed that P2ry12 is a unique microglial molecule that acts as a marker of resident homeostatic microglia in the CNS [33]. Furthermore, studies modelling CNS injury have reported a dramatic reduction in P2ry12 microglial expression, which suggests that microglia lose major homeostatic functions following CNS insults [69, 70]. The observed reduction in P2ry12+ microglia (in addition to astrocyte activation) in our CIPN model suggests that treatment with PTX and OXA may induce pathological changes in the spinal cord, thereby affecting central pain processing.

Upon examination of cytokine and chemokine levels on day 13 post-1^st^ injection in the spinal cord, we found that levels of TNF-α, IFN-γ, CCL11, CCL4, CCL3, IL-12p70 and GM-CSF were significantly upregulated in the PTX-treated group compared to saline. Although there were some subtle changes, OXA did not cause significant changes in cytokine or chemokine levels in the spinal cord. Cytokine dysregulation in the CNS has been implicated in many neuropathic pain conditions, and has been directly linked to the activation of nociceptive neurons and stimulation of pain hypersensitivity [71–73]. Cytokines can act as both stimulators and mediators of glial function (astrocytes and microglia), and interaction of different cytokines can activate distinct functional processes in glia-mediated signalling during and post injury [71, 74, 75]. Since there were no changes in innate or adaptive immune cell populations in the spinal cord, cytokine upregulation in the spinal cord of PTX-treated animals is likely driven by central terminals of damaged neurons, activated astrocytes, or through other unknown mechanisms. Furthermore, the lack of astrocyte activation in the spinal cord of OXA-treated mice may explain the absence of alterations in the profile of spinal cytokines and chemokines.

## Conclusions

In conclusion, this study demonstrates that although both PTX and OXA treatment induce pain hypersensitivity, these drugs have differential effects on peripheral immunity and neuroinflammation. Specifically, both PTX and OXA were associated with an increase in CD4+ and CD8+ T-cells in the blood. However, only OXA caused a significant reduction in lymph node T-reg cells, and further depletion of these immunosuppressive cells had no effect on pain hypersensitivity. PTX selectively caused marked phenotypic changes in both the peripheral and central nervous systems, characterised by IB4+ and NF200+ sensory neuronal injury in the DRG, and astrocyte activation and cytokine/chemokine changes in the spinal cord. In addition, both PTX and OXA induced a significant reduction in spinal P2ry12+ resident microglia. As the mechanisms of action of PTX and OXA are different, it is not surprising that they induce distinctive effects on the immune and nervous systems with varying degrees of neuroinflammation. Further studies are required to investigate the potential role of these changes in stimulating or maintaining CIPN and associated neuropathic pain.

## Supporting Information

S1 Fig Systemic inflammatory changes in the inguinal lymph nodes following chemotherapy. Flow cytometry of lymphocytes to characterise inflammatory changes in the inguinal lymph nodes were carried out on days 7 and 13 post-1^st^ paclitaxel (PTX), oxaliplatin (OXA) or saline (control) injection. Column graphs of CD4+ cells (A, E) and total intracellular IFN-γ (B, F), IL-17α (C, G) and IL-4 (D, H) cytokine levels, expressed as percentages of lymphocyte singlets. No significant changes detected in CD4+ cell population and IFN-γ, IL-17α and IL-4 positive lymphocytes in PTX- and OXA- treated mice compared with saline controls on day 7 (n = 10) and day 13 (n = 4–10). One-way ANOVA followed by Bonferroni's multiple comparison’s test. Data expressed as mean±SEM.
(TIF)

S2 Fig Systemic inflammatory changes in the spleen following chemotherapy. Flow cytometry of lymphocytes to characterise inflammatory changes in the spleen were carried out on days 7 and 13 post-1^st^ paclitaxel (PTX), oxaliplatin (OXA) or saline (control) injection. Column graphs of CD4+ cells (A, E), CD4+CD25+FoxP3+ regulatory T-cells (T-regs) (D), and IFN-γ (B, F), IL-17α (C, G) and IL-4 (H) positive lymphocytes, expressed as percentages of lymphocyte singlets. No significant changes were detected in CD4+ and T-reg cell populations, and in IFN-γ and IL-17α positive lymphocytes in PTX- and OXA-treated mice compared with saline controls on day 7 (n = 6–9) and day 13 (n = 3–10). No significant changes were detected in IL-4 cytokine levels on day 13 (n = 3–8). One-way ANOVA followed by Bonferroni's multiple comparison’s test. Data expressed as mean±SEM.
(TIF)

S3 Fig Systemic inflammatory changes in the blood following chemotherapy. Flow cytometry of lymphocytes was carried out in the blood on day 13 post-1^st^ paclitaxel (PTX), oxaliplatin (OXA) or saline (control) injection. Column graphs of CD45+CD4+ cells (A) and CD45+CD8+ cells (B), expressed as percentages of lymphocyte singlets. No significant changes were seen in CD45+CD4+ and CD45+CD8+ cell populations in PTX- and OXA-treated mice compared with saline controls (n = 10). One-way ANOVA followed by Bonferroni's multiple comparison’s test. Data expressed as mean±SEM.
(TIF)

S4 Fig Inflammatory changes in the spinal cord and lumbar DRG following chemotherapy. Flow cytometry of lymphocytes to characterise inflammatory changes in spinal cord and L3-L4 DRGs were carried out on day 13 post-1^st^ paclitaxel (PTX), oxaliplatin (OXA) or saline (control) injection. Column graphs of CD45+CD4+ cells (A) and CD45+CD8+ cells (B) in the DRGs, and CD45+CD4+ cells (C) and CD45+CD8+ cells (D) in the spinal cord, expressed as percentages of CD45+ lymphocyte singlets. No significant changes were seen in CD45+CD4+ and CD45+CD8+ cell populations in DRG or spinal cord of PTX-and OXA-treated mice compared with saline controls (n = 5–6). One-way ANOVA followed by Bonferroni's multiple comparison’s test. Data expressed as mean±SEM.
(TIF)

S1 Table Cytokine and chemokine changes in serum following chemotherapy. Bio-plex analysis of cytokine and chemokine profile was carried out in serum on day 13 post-1^st^ injection of paclitaxel (PTX), oxaliplatin (OXA) or saline (control). Table showing median and interquartile range of cytokine/chemokine concentrations (pg/ml) in the serum diluted 1:4 (n = 5). There are no significant differences between treatment groups; Kruskal Wallis test followed by Dunn’s multiple comparisons test.
(TIF)

S2 Table Raw data of Figs 1–8. (XLSX)

S3 Table Raw data of supplementary figures S1–S4 Figs. (XLSX)
