# Supplementary figures and images for: Characterisation of Immune and Neuroinflammatory Changes Associated with Chemotherapy-Induced Peripheral Neuropathy
Source: PLoS One. 2017 Jan 26;12(1):e0170814. doi: 10.1371/journal.pone.0170814 (PMC5268425; doi:10.1371/journal.pone.0170814)

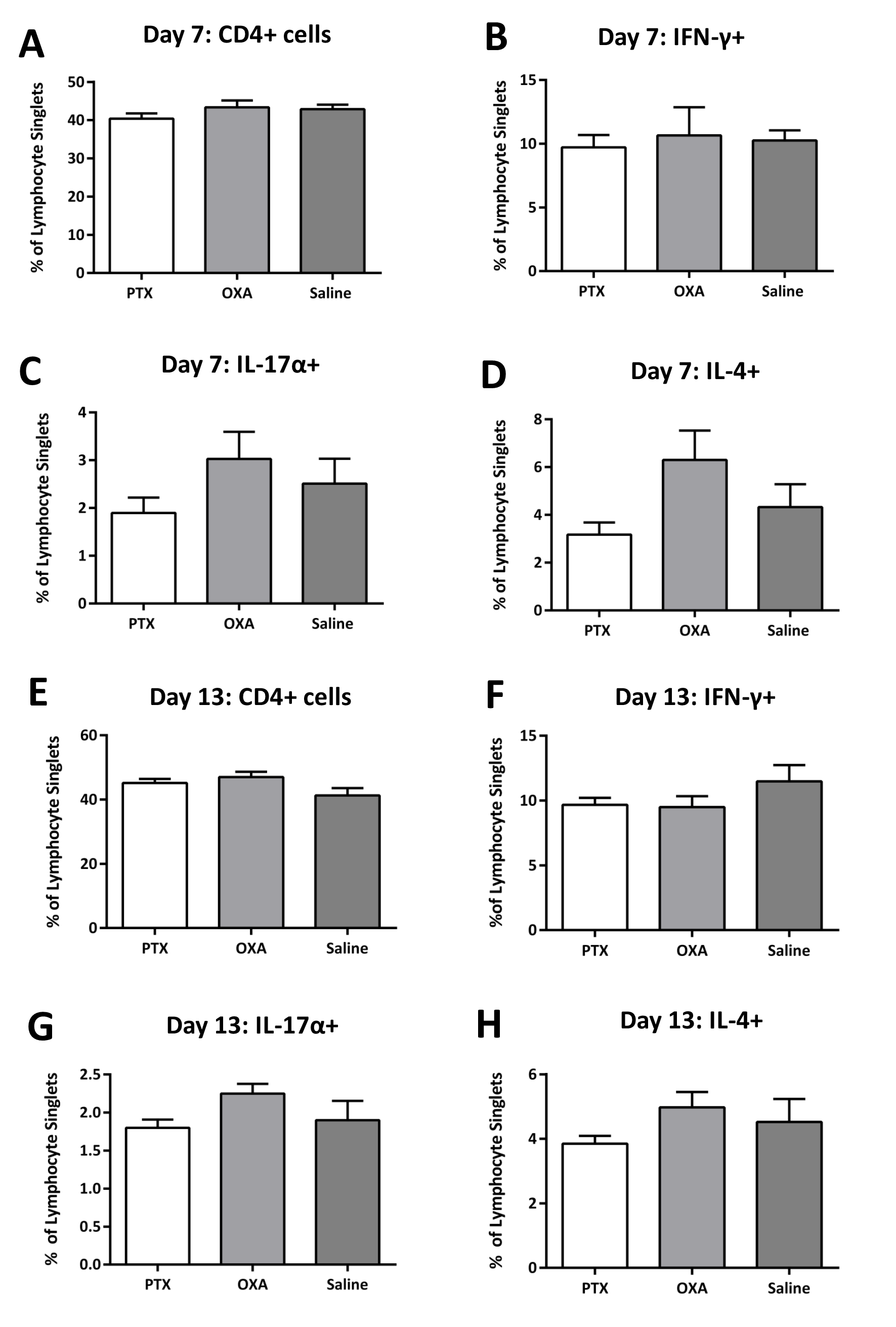

Supplement: S1 Fig — Flow cytometry of lymphocytes to characterise inflammatory changes in the inguinal lymph nodes were carried out on days 7 and 13 post-1st paclitaxel (PTX), oxaliplatin (OXA) or saline (control) injection. Column graphs of CD4+ cells (A, E) and total intracellular IFN-γ (B, F), IL-17α (C, G) and IL-4 (D, H) cytokine levels, expressed as percentages of lymphocyte singlets. No significant changes detected in CD4+ cell population and IFN-γ, IL-17α and IL-4 positive lymphocytes in PTX- and OXA- treated mice compared with saline controls on day 7 (n = 10) and day 13 (n = 4–10). One-way ANOVA followed by Bonferroni's multiple comparison’s test. Data expressed as mean±SEM. (TIF) [file pone.0170814.s001.tif]

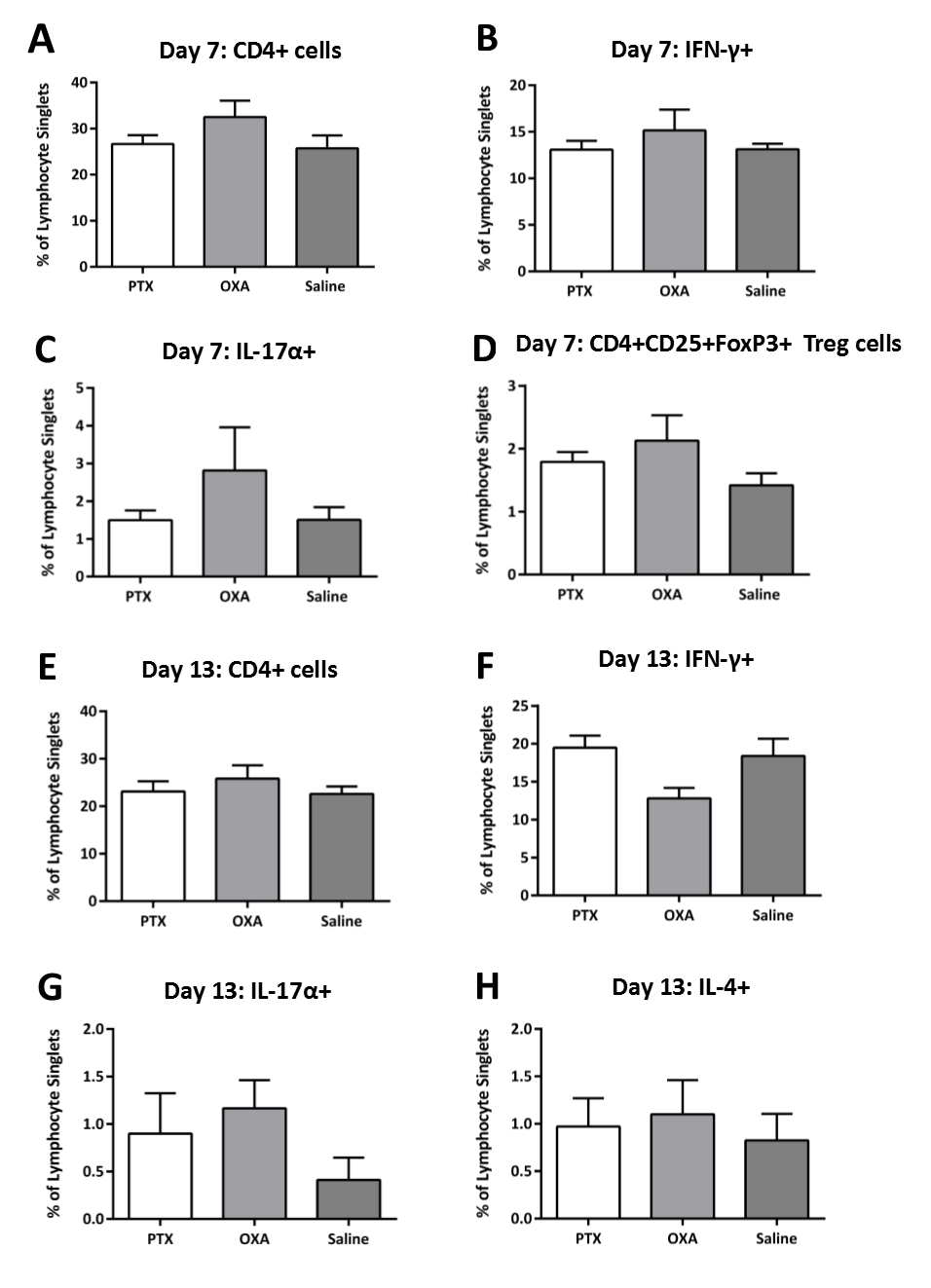

Supplement: S2 Fig — Flow cytometry of lymphocytes to characterise inflammatory changes in the spleen were carried out on days 7 and 13 post-1st paclitaxel (PTX), oxaliplatin (OXA) or saline (control) injection. Column graphs of CD4+ cells (A, E), CD4+CD25+FoxP3+ regulatory T-cells (T-regs) (D), and IFN-γ (B, F), IL-17α (C, G) and IL-4 (H) positive lymphocytes, expressed as percentages of lymphocyte singlets. No significant changes were detected in CD4+ and T-reg cell populations, and in IFN-γ and IL-17α positive lymphocytes in PTX- and OXA-treated mice compared with saline controls on day 7 (n = 6–9) and day 13 (n = 3–10). No significant changes were detected in IL-4 cytokine levels on day 13 (n = 3–8). One-way ANOVA followed by Bonferroni's multiple comparison’s test. Data expressed as mean±SEM. (TIF) [file pone.0170814.s002.tif]

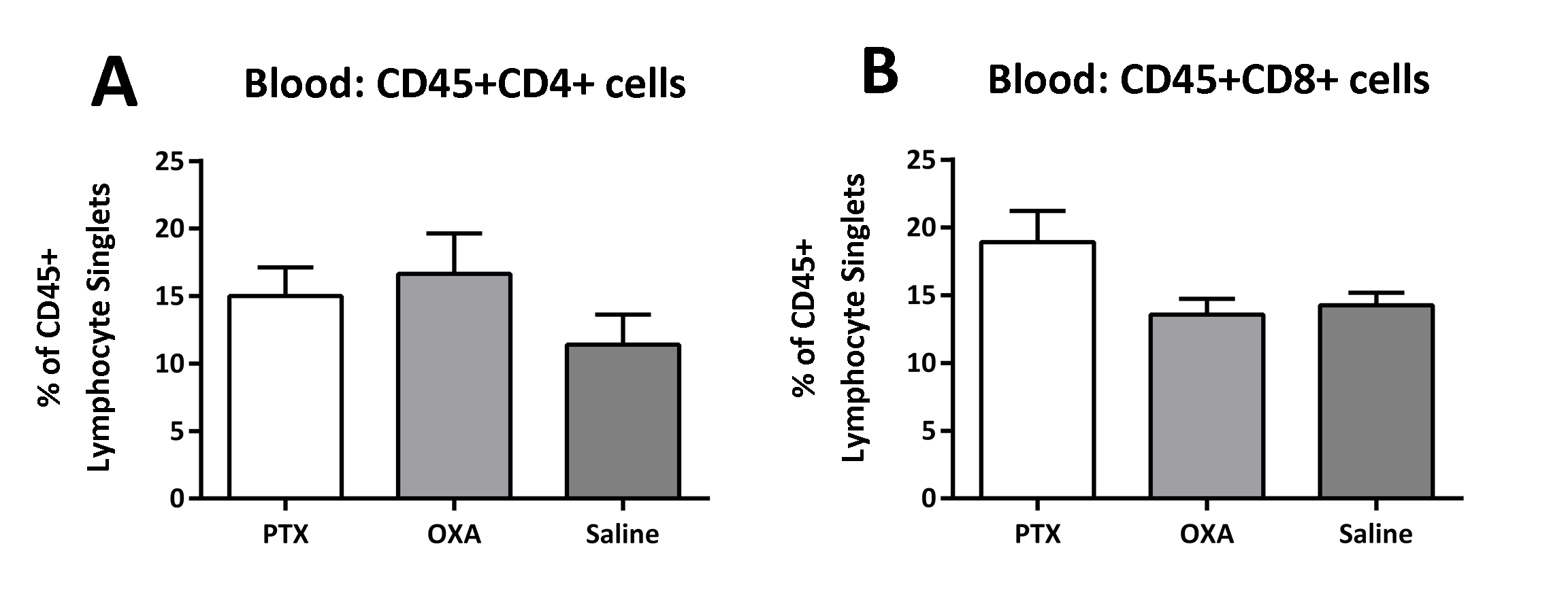

Supplement: S3 Fig — Flow cytometry of lymphocytes was carried out in the blood on day 13 post-1st paclitaxel (PTX), oxaliplatin (OXA) or saline (control) injection. Column graphs of CD45+CD4+ cells (A) and CD45+CD8+ cells (B), expressed as percentages of lymphocyte singlets. No significant changes were seen in CD45+CD4+ and CD45+CD8+ cell populations in PTX- and OXA-treated mice compared with saline controls (n = 10). One-way ANOVA followed by Bonferroni's multiple comparison’s test. Data expressed as mean±SEM. (TIF) [file pone.0170814.s003.tif]

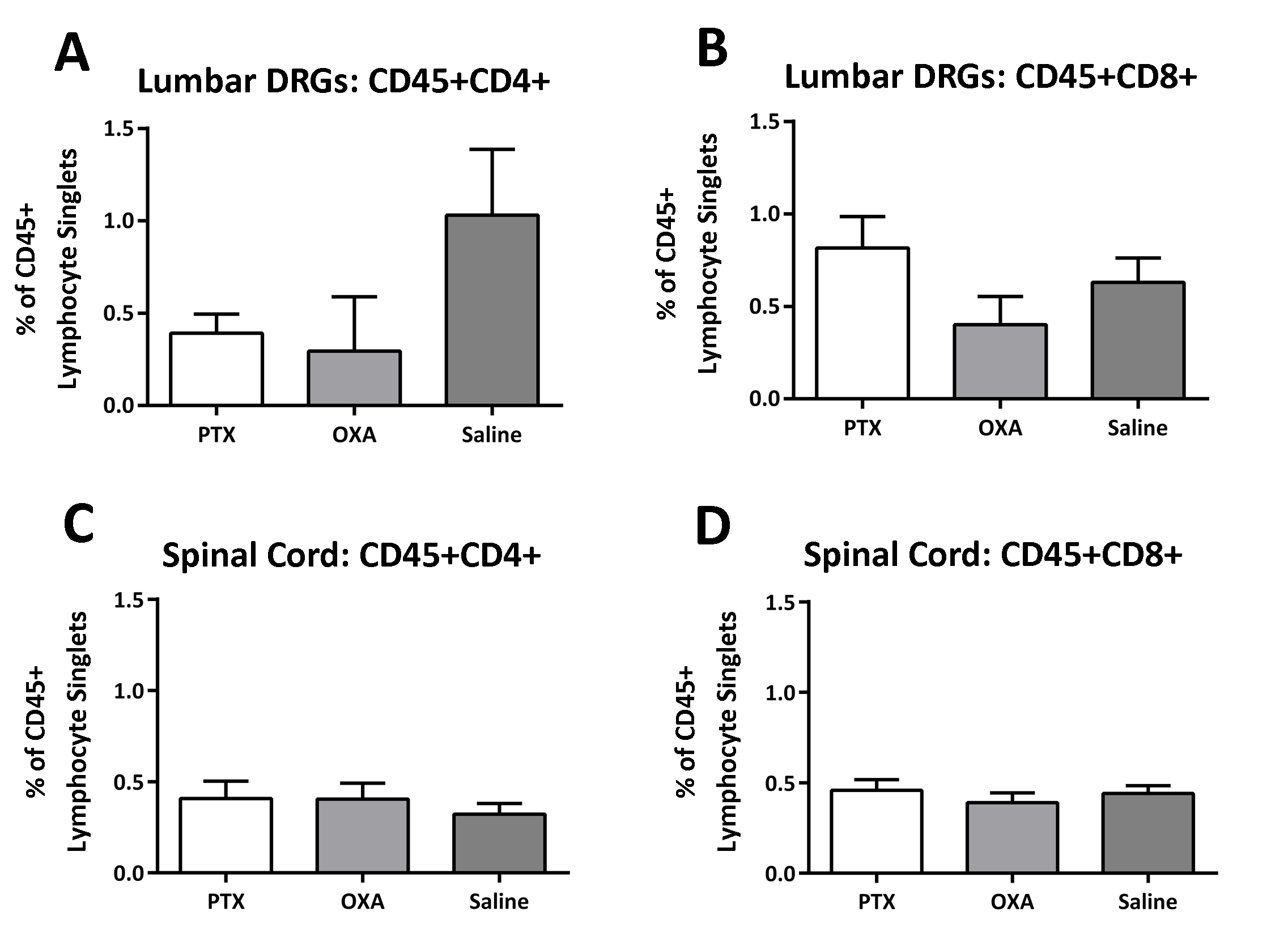

Supplement: S4 Fig — Flow cytometry of lymphocytes to characterise inflammatory changes in spinal cord and L3-L4 DRGs were carried out on day 13 post-1st paclitaxel (PTX), oxaliplatin (OXA) or saline (control) injection. Column graphs of CD45+CD4+ cells (A) and CD45+CD8+ cells (B) in the DRGs, and CD45+CD4+ cells (C) and CD45+CD8+ cells (D) in the spinal cord, expressed as percentages of CD45+ lymphocyte singlets. No significant changes were seen in CD45+CD4+ and CD45+CD8+ cell populations in DRG or spinal cord of PTX-and OXA-treated mice compared with saline controls (n = 5–6). One-way ANOVA followed by Bonferroni's multiple comparison’s test. Data expressed as mean±SEM. (TIF) [file pone.0170814.s004.tif]

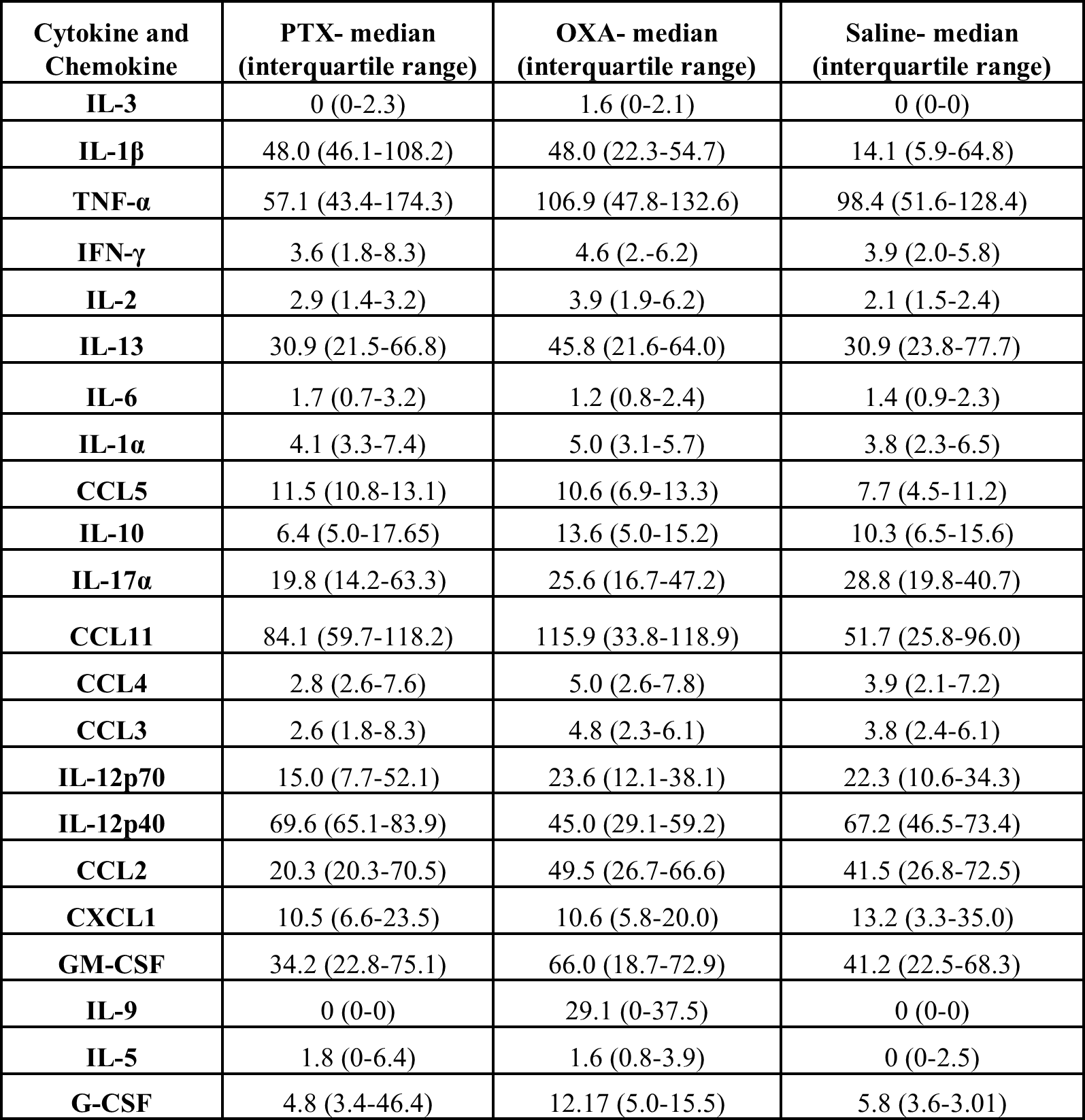

Supplement: S1 Table — Bio-plex analysis of cytokine and chemokine profile was carried out in serum on day 13 post-1st injection of paclitaxel (PTX), oxaliplatin (OXA) or saline (control). Table showing median and interquartile range of cytokine/chemokine concentrations (pg/ml) in the serum diluted 1:4 (n = 5). There are no significant differences between treatment groups; Kruskal Wallis test followed by Dunn’s multiple comparisons test. (TIF) [file pone.0170814.s005.tif]
